# Are gut dysbiosis, barrier disruption, and endotoxemia related to adipose tissue dysfunction in metabolic disorders? Overview of the mechanisms involved

**DOI:** 10.1007/s11739-023-03262-3

**Published:** 2023-04-04

**Authors:** Daniela Rosendo-Silva, Sofia Viana, Eugénia Carvalho, Flávio Reis, Paulo Matafome

**Affiliations:** 1 Faculty of Medicine, Coimbra Institute for Clinical and Biomedical Research (iCBR), University of Coimbra, Coimbra, Portugal; 2 Institute of Physiology, Faculty of Medicine, University of Coimbra, Coimbra, Portugal; 3 Center for Innovative Biomedicine and Biotechnology (CIBB), University of Coimbra, Coimbra, Portugal; 4 Clinical Academic Center of Coimbra (CACC), Coimbra, Portugal; 5 Institute of Pharmacology and Experimental Therapeutics, Faculty of Medicine, University of Coimbra, Coimbra, Portugal; 6 Instituto Politécnico de Coimbra, Coimbra Health School (ESTeSC), Coimbra, Portugal; 7 Center of Neuroscience and Cell Biology (CNC), University of Coimbra, Coimbra, Portugal; 8 Institute for Interdisciplinary Research, University of Coimbra, Coimbra, Portugal; 9 Faculty of Medicine, Pole III of University of Coimbra, Subunit 1, 1st floor, Azinhaga de Santa Comba, Celas, 3000-354 Coimbra, Portugal

**Keywords:** Gut dysbiosis, Intestinal permeability, Endotoxemia, Adipose tissue microbiota, Metabolic disease

## Abstract

Recently, compelling evidence points to dysbiosis and disruption of the epithelial intestinal barrier as major players in the pathophysiology of metabolic disorders, such as obesity. Upon the intestinal barrier disruption, components from bacterial metabolism and bacteria itself can reach peripheral tissues through circulation. This has been associated with the low-grade inflammation that characterizes obesity and other metabolic diseases. While circulating bacterial DNA has been postulated as a common feature of obesity and even type 2 diabetes, almost no focus has been given to the existence and effects of bacteria in peripheral tissues, namely the adipose tissue. As a symbiont population, it is expected that gut microbiota modulate the immunometabolism of the host, thus influencing energy balance mechanisms and inflammation. Gut inflammatory signals cause direct deleterious inflammatory responses in adipose tissue and may also affect key gut neuroendocrine mechanisms governing nutrient sensing and energy balance, like incretins and ghrelin, which play a role in the gut-brain-adipose tissue axis. Thus, it is of major importance to disclose how gut microbiota and derived signals modulate neuroendocrine and inflammatory pathways, which contribute to the dysfunction of adipose tissue and to the metabolic sequelae of obesity and related disorders. This review summarizes the current knowledge regarding these topics and identifies new perspectives in this field of research, highlighting new pathways toward the reduction of the inflammatory burden of metabolic diseases.

## Introduction

The gastrointestinal (GI) tract is classically perceived as the main responsible for digestion, allowing nutrient absorption and fluids/electrolytes balance. Nonetheless, the gut orchestrates a plethora of other relevant functions, such as regulation of energy homeostasis, immune sensing, and protection from external harmful factors [1].

Energy balance is a delicate process relying on the rhythmic alignment between the central nervous system (CNS) and peripheral informants that sense nutrient arrival and scarcity, i.e., the gut, the pancreas, and the adipose tissue (AT) [2]. The energetic status, communicated to brain centers through neuronal and peripheral humoral effectors, will determine an appropriate response to adapt food intake, nutrient partitioning, and energy expenditure (catabolism of stored lipids and thermogenesis) accordingly [2, 3].

The unique anatomy of the GI tract, especially in the intestines, allows it to work as an important physical, but selective, wall of defense against external pathogens, while maintaining neutrality to gut commensals. The intestinal mucosal barrier is composed of different layers: the lumen, mucus layer, intestinal epithelium, and the lamina propria [reviewed by Farré et al*.* [1] and Vancamelbeke et al*.* [4]]. The outer mucus layer allocates a huge diversity of commensal microorganisms, known as the gut microbiota, and respective metabolites, as well as antimicrobial peptides. The mucus layer is mainly composed of water and mucins (glycosylated proteins secreted by goblet cells) and confers a semi-permeable environment, allowing the passage of factors from the lumen to the epithelial cells, while preventing the extravasation of microorganisms. An intricate relationship occurs between gut microbiota and the mucus gel since the antimicrobial peptides repel microorganisms that, in turn, can influence the gel composition [1, 4, 5]. In fact, the mucus phenotype is transmissible through fecal microbiota transplantation into germ-free mice, which acquire mucosal properties dependent on the microbiota of the donor [5]. Different types of epithelial cells constitute the intestinal epithelium in a single-layer structure that divides the lumen from the lamina propria. The enterocytes are the cells responsible for maintaining structural integrity and regulating absorption; the goblet cells secrete mucus; Paneth cells produce the antimicrobial peptides; M cells are involved in immunity responses, and the enteroendocrine cells produce hormones and neurotransmitters, allowing communication between the gut and several organs [1, 4, 6]. A rupture in the mucosal or epithelial barriers can compromise the gut-vascular interface (endothelium, pericytes, and enteric glial cells), allowing pathogens and bacterial products to disseminate mainly through the portal vein circulation to peripheral sites, such as the liver or visceral fat [7]. Thus, the gut barrier exists as a dynamic structure that regulates metabolic homeostasis, whose function is compromised in metabolic diseases such as obesity, metabolic-associated fatty liver disease (MAFLD), and type 2 diabetes (T2DM) [7–10].

In this review, we will highlight the contribution of gut inflammatory signals (endotoxemia) and bacteria itself on AT dysfunction in obesity, a main factor for the development of other metabolic disorders. We will briefly review the process of diet-driven dysbiosis, intestinal barrier disruption and metabolic endotoxemia (1), and will deeply cover two main points: (2) the consequences of dysbiosis and intestinal inflammation on gut neuroendocrine function and (3) the deleterious effects of impaired gut function on AT.

1. Diet-driven dysbiosis, gut inflammation, and intestinal barrier disruption

Trillions of microorganisms, most of them bacteria, inhabit the human gut in a symbiotic relationship with the host and this colonization is a dynamic and ongoing process throughout life. The gut microbiota plays an active part in the regulation of (1) host defenses, by training the immune system through pathogen-associated molecular patterns (PAMPs) and antigens exposure [11]; (2) digestion, as dietary indigestible products are degraded by bacteria [12]. Indeed, gnotobiology studies in germ-free mice show a deficient immune response, originating from morphological and functional malformations, insufficient mesenteric lymph nodes, and incapacity to produce antibodies [11]. During digestion, gut microbiota produce short-chain fatty acids (SCFAs) from the metabolism of dietary fibers, which are important not only to maintain the epithelial barrier but also to keep immune homeostasis [13]. SCFAs are correlated with better glycemic and lipid profiles and their contribution to improved metabolism arises from direct action on peripheral sites, such as AT (decrease lipolysis), liver (increase fatty acid oxidation), and pancreas (potentiate glucose-stimulated insulin secretion) [14, 15]. Other common bacterial metabolites are trimethylamine (derived from choline and carnitine), lipopolysaccharide (LPS) (derived from the Western diet), indole (derived from tryptophan), and p-cresol (derived from tyrosine) [7].

**Fig. 1:**
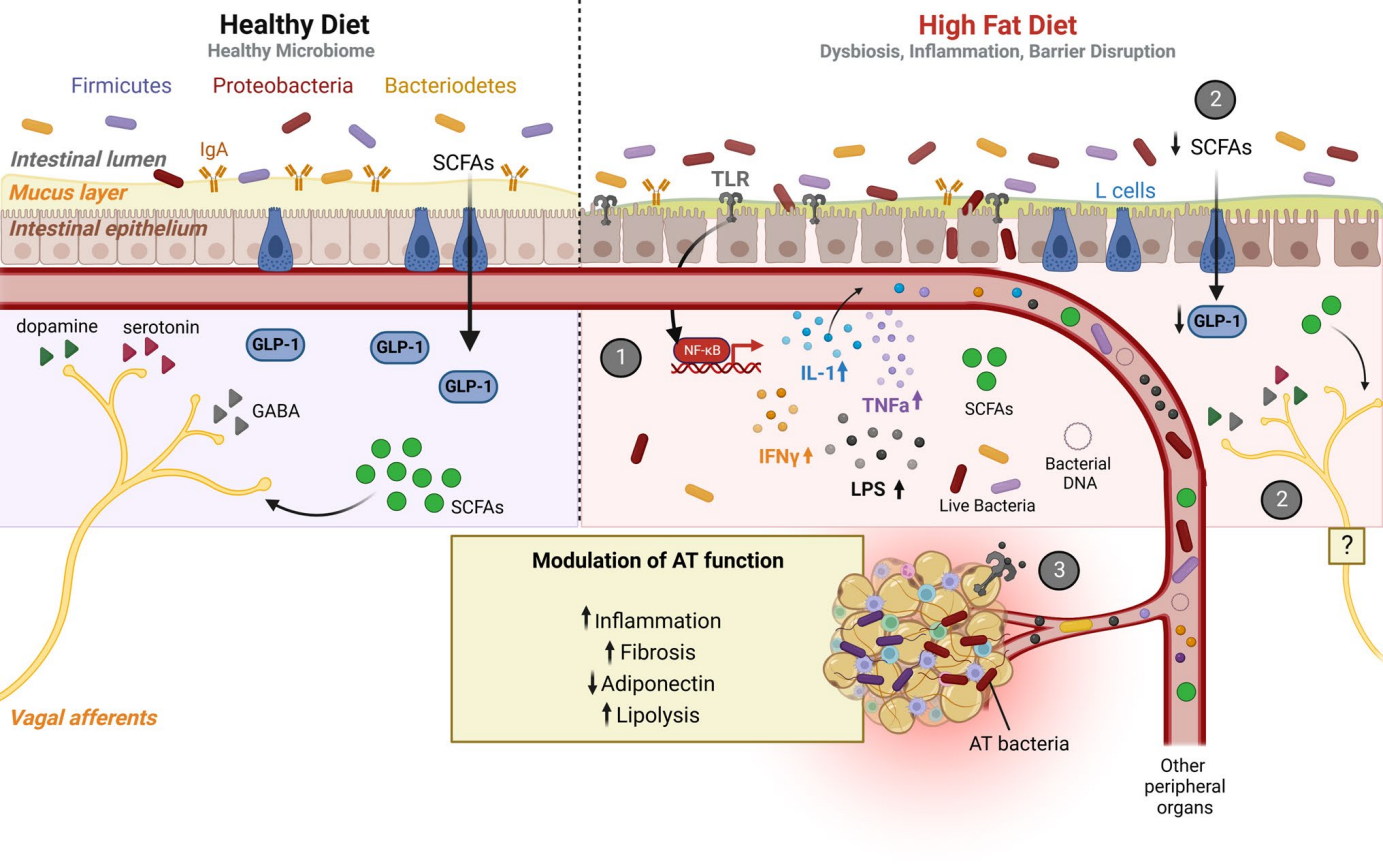
The putative mechanisms linking HF diet-driven dysbiosis to impaired AT function. HFD initiates a loop of gut dysbiosis and inflammation, dampening epithelial barrier integrity, and thus increasing intestinal permeability. Because of dysbiosis, LPS levels rise while SCFAs production is impaired. **1**—LPS-meditated TLR activation triggers NF-kB-dependent transcription programs for pro-inflammatory cytokines: IL-1, TNFα, and IFNy, contributing to colonic inflammation, that may spread into the circulation. **2**—A reduction in the SCFAs production hinders GLP-1 secretion by L cells. Additionally, since SCFAs, mainly butyrate, directly stimulate vagal afferents in healthy conditions (left side), a decrease in the SCFAs production will likely result in decreased vagal tone, which might compromise the gut-brain axis and energy balance regulation (right side). **3**—Increased intestinal permeability facilitates LPS, cytokines, and bacteria translocation and migration to distant peripheral targets, via the circulation. Colonization of AT by gut-derived bacteria and LPS-mediated TLR activation will culminate in an inflammatory response and overall impairment of AT function. Abbreviations: *SCFAs* short-chain fatty acids, *GLP-1* glucagon-like peptide 1, *GABA* y-aminobutyric acid, *TLR* toll-like receptor, *NF-kB* nuclear factor kappa-light-chain-enhancer of activated B cells, *IL-1* interleukin 1, *IFNy* interferon-gamma, *LPS* lipopolysaccharide, *TNFa* Tumor necrosis factor-alpha, *AT* adipose tissue. Image created with Biorender.com

An adequate balance between the main bacterial phyla (*Bacteroidetes, Firmicutes, Actinobacteria, Proteobacteria, Verrucomicrobia*) and the overall diversity of bacterial groups, as well as a healthy ratio of symbionts/pathobionts, should be maintained to avoid dysbiosis, which facilitates a disease-promoting environment [15]. Well-known contributors to dysbiosis are, to name a few, antibiotics, drugs, alcohol, stress, genetic susceptibility, and diet, in particular, the westernized high-fat diets (HFD) (reviewed by Weiss and Hennet) [15–17]. The first studies emerging on the link between diet-induced obesity and a shift in gut microbiota were performed by Gordon and co-workers [18, 19] (Table 1). Further, gut microbiota was shown to be an active part of fat storage regulation [20]. In fact, in C57BL/6 J mice, HFD consumption for 4 weeks, was sufficient to induce marked alterations in gut flora composition [21–24], and fecal transplantation to germ-free mice induced a massive increase in adiposity [19]. Similar effects were observed when diets were extended to 8, 12, or 16 weeks [25–27] (Table 1), meaning that a short period is enough to induce such marked alteration in gut flora and structure. Diet-induced obesity promotes dysbiosis mainly by increasing *Firmicutes/Bacteroidetes* ratio, in mice and rats, with an overall loss in genus diversity [15, 17, 21–32] (Table 1), which has been shown to promote the increased ability for energy accretion from food and generation of an inflammatory environment [33]. In an HFD-driven dysbiosis environment, with a high prevalence of LPS-producing Gram-negative bacteria, Toll-like receptors (TLRs) may be continuously activated, namely TLR4. This will promote downstream activation of nuclear factor kappa β (NF-ĸB)-dependent transcription programs for several pro-inflammatory cytokines -, interleukin (IL)-1β, IL-18, IL-6, IL-33, tumor necrosis factor α (TNFα) and interferon-gamma (IFNy), eventually contributing to colonic inflammation [25, 26, 28, 31, 32, 34, 35] (Fig. 1(1)). Yet, decreased amounts of T_H_17 cells and eosinophils have been reported in the small intestine of HFD-fed mice, during 1 to 4 weeks of diet, which are features of a reduced intestinal inflammatory process [23, 36] (Table 1). Moreover, no histological hallmarks of intestinal inflammation (e.g., mononuclear cell infiltrate, epithelial hyperplasia, or goblet cell depletion) were observed in most studies even when molecular markers of inflammation were present and under chronic regimens of HFD feeding [28, 36]. Albeit still controversial, sustained activation of inflammatory pathways, which appear to be mainly triggered by TLR4 activation [25], may perpetuate a chronic inflammation state, causing damage to the epithelial barrier (Fig. 1(1)). In fact, HFD-induced gut dysbiosis and inflammation compromise barrier integrity by decreasing tight-junction proteins, such as zonulin, occludin, and claudin-1/5 [25, 26, 29, 41] (Table 1). The influence of dysbiosis is clearly proven by antibiotic treatment, which indeed prevented diet-induced barrier breakage [22].

**Table 1:** The putative relationship between diet-driven dysbiosis, gut inflammation, and intestinal barrier disruption with adiposity, in animal models and human studies

Model/protocol	Gut microbiota (phylum/class, order, family or genus)	Gut inflammation	Intestinal permeability & endotoxemia	Body adiposity	References
C57BL/6 J mice/HF diet feeding (1 week)	N/A	↓ TNF-α, MCP-1, = CD11b, CD11c, IL-1β mRNA ↓ eosinophils No histological features of inflammation	↑ FITC-dextran	N/A	[36]
C57BL/6 J mice/HF diet feeding (4 weeks)	*↓ Bacteroidetes* *↓* *Actinobacteria/Bifidobacterium*	N/A	↓ colonic zonulin and occludin ↑ FITC-dextran ↑ LPS (plasma)	↑	[21, 22]
	*↓Bacteroidetes/Porphyromonadaceae* ↑ *Firmicutes/Peptostreptococcaceae*	↓ IL-22, IL-17, IL-10 mRNA and *↓*Th17 cells	N/A	↑	[23]
	*↓ Bacteroidetes* ↑ *Firmicutes/Mollicutes*	N/A	N/A	↑	[24]
C57BL/6 J mice/ HF diet feeding (8 weeks)	*↓ Bacteroidetes* ↑ *Firmicutes/Ruminococcaceae* *↓ Proteobacteria*	↑ TNFa, IL-1β, IL-6, TLR4 and NF-kB mRNA	↑ LPS (plasma) ↓ colonic claudin-1 and occludin	↑	[25]
C57BL/6 J mice/HF diet feeding (12 weeks)	↑ *Firmicutes/Oscillibacter* *↓ Firmicutes/Lactobacillus* *↓ Bacteroidetes*	↑ TNF-α, = IL-6 mRNA	↓ transepithelial resistance ↓ colonic zonulin	↑	[26]
	N/A	N/A	↑ FITC-dextran ↓ colonic occludin and claudin-5 recruitment of immune cells into the intestinal epithelial layer (H&E staining)	↑	[41]
C57BL/6 J mice/HF diet feeding (16 weeks)	N/A	↑ ileum NF-kB, TNF-α	N/A	↑	[34]
	*↓ Bacteroidetes* ↑ *Firmicutes*	N/A	N/A	↑	[27]
C57BL/6 J mice/HF diet feeding (80 weeks)	*↓ Bacteroidetes* ↑ *Firmicutes* *↓ Tenericutes/Anaeroplasma* ↑ *Actinobacteria/Adercreutzia*	↑ IL-6, = MCP-1, IL-10, IL-17 mRNA No histological features of inflammation	N/A	↑	[28]
C57BL/6 J mice/HS or HFr diet feeding (12 weeks)	↑ *Firmicutes* ↑ *Proteobacteria/Desulfovibrio* *↓ Bacteroidetes/Muribaculum* ↑ Verrucomicrobia/*Akkermansia*	↑ IL-1β, TNF-α mRNA	↑ LPS (plasma) ↑ FITC-dextran ↓ colonic zonulin and occludin	↑	[29]
GF C57BL/6 J mice/fecal transplantation from HF/HS-fed donors	↑ *Firmicutes/Erysipelotrichi* ↑ *Firmicutes/Bacilli* *↓* *Bacteroidetes*	N/A	N/A	↑	[19]
Sprague–Dawley rats/ HF diet feeding (12 weeks)	*↓ Bacteroidetes* ↑ *Firmicutes/Clostridiales* *↓ Firmicutes/Lactobacillus* *↓Tenericutes*	N/A	N/A	N/A	[30]
	↑ *Proteobacteria/Enterobacteriales* ↑ *Firmicutes/Clostridiales* = *Bacteroidetes/Bacteroidales*	↑ ileum TLR4/MD2	↑ LPS (plasma) ↑ FITC-dextran	↑	[31]
Sprague–Dawley rats/HF-HS diet feeding (4 weeks)	*↓ Bacteroidetes* ↑ *Firmicutes* ↑ *Actinobacteria/Micrococcaceae* ↑ *Tenericutes/Anaeroplasmatales*	↑ IL-6, IL-1β, TNF-α mRNA	↑ LPS (plasma) ↓ cecum occludin	↑	[32]
Adults with obesity	N/A	N/A	↑ LPS (plasma)	N/A	[44]
	↑ *Firmicutes* *↓ Bacteroidetes*	N/A	N/A	N/A	[45]
	*↓* *Actinobacteria/Bifidobacterium*	= fecal calprotectin	= lactulose/mannitol = lactulose/sucralose	N/A	[48]
Women with overweight or obesity	N/A	N/A	↑ lactulose and sucralose/mannitol excretion	N/A	[46, 47]
Healthy adults on HF diet (5 days)	↑ *Firmicutes/Clostridiales* ↑ *Proteobacteria/Bilophila*	N/A	N/A	N/A	[49]
Women with obesity on a low-calorie diet (14 days)	↓ *Firmicutes/Agathobacter* ↑ *Actinobacteria/Bifidobacterium*	N/A	↓ lactulose and sucralose excretion = mannitol excretion ↓ plasma zonulin and LBP	↓	[51]

*N*/A Not applicable or not measured, *HF* high-fat, *HS* high-sugar, *HFr* high-fructose, *LPS* lipopolysaccharide, *GF* germ-free*, MCP-1* monocyte chemoattractant protein-1, *NF-kB* nuclear factor kappa β, *TLR4/MD2* Toll-like receptor 4/myeloid differentiation protein-2, *FITC* fluorescein isothiocyanate, *IL* interleukin, *TNFα* tumor necrosis factor alpha, *LBP* lipopolysaccharide-binding protein

Another mechanism possibly involved in the regulation of barrier integrity is the endocannabinoid system, which is highly upregulated by HFD consumption [37]. Cannabinoid receptor 1 has been shown to modulate barrier permeability. Rimonabant administration to *ob/ob* mice reduces intestinal permeability (measured by circulating LPS levels), whereas agonism displayed differential effects, leading to increased (with anandamide) or decreased (with 2-arachidonoylglycerol) barrier integrity (reviewed by Cuddihey et al*.*) [38].

HFD is also associated with increased hepatic bile acids (BA) secretion, which are converted to secondary BA by the microbiota within the colon. Secondary BA play a role in fat digestion and can in turn control the gut microbiome (increasing *Firmicutes/Bacteroidetes* ratio*)* while reducing the risk of intestinal inflammation [7]. However, at chronically high levels, secondary BA are associated with colonic inflammation and intestinal barrier dysfunction [7, 39, 40]. A 2-week HFD regimen, in mice, potentiated secondary BA production by increasing *Lachnospiraceae* and *Rumincoccaceae (Firmicutes/Clostridiales),* which deconjugate and transform the liver-derived primary BA into secondary intestinal BA [39] while inducing gut epithelium proliferation, which may arise as an initial protective mechanism.

Furthermore, a 4-week HFD, in C57BL6/J mice, was already able to increase LPS levels in circulation, inducing LPS-dependent systemic inflammation, the so-called metabolic endotoxemia, which might initiate/contribute to the metabolic sequelae of obesity [21]. The relevance of impaired barrier integrity as a pathological mechanism for obesity (reviewed by Portincasa et al*.*) [7] was recently further validated, since extracellular vesicles from *Akkermansia muciniphila* (*Verrucomicrobia*) were able to reduce weight gain of HFD-fed mice, by improving tight-junctions, and thus decreasing intestinal permeability, through an AMP-activated protein kinase (AMPK)-dependent mechanism [41].

Glucose and fructose-enriched diets were also shown to induce loss in tight-junction proteins and increased gut barrier permeability assayed by plasma LPS levels and fluorescein isothiocyanate (FITC)-dextran administration, although to a lower extent than after the HFD feeding [29] (Table 1). Refined sugars, such as saccharides, are a typical component of Western diets and were linked to TLR4-mediated colonic inflammation and increased permeability [42]. Hyperglycemia enhanced glucose transporter 2 (GLUT2)-mediated retrograde glucose uptake into epithelial cells, leading to increased N-glycan biosynthesis, also dampening barrier integrity [43].

Although westernized diets (high sugar and HFD) are associated with a pattern of dysbiosis (increased *Firmicutes/Bacteroidetes* ratio), putative intestinal inflammatory response, and barrier disruption in rodents (Table 1), this link is less evident in humans, in part because it is harder to study. However, some studies in humans have already shown a relationship between HFD-feeding and/or obesity scenarios with alterations in gut colonization and intestinal barrier disruption (Table 1). The *Firmicutes/Bacteroidetes* ratio and circulating LPS levels (7.8 EU/mL) are increased in patients with obesity when compared to normal-weighted ones [44, 45] (Table 1). However, such measurements are merely indirect indicators of barrier integrity, since the definition of the normal range of values is uncertain. The lactulose/mannitol or sucralose/mannitol excretion ratios are more useful to assess intestinal permeability, allowing to discriminate between small intestinal permeability (lactulose/mannitol) or colonic permeability (sucralose/mannitol) [4]. In women with obesity, the permeability of the small intestine was increased, as measured by the lactulose/mannitol excretion, and correlated positively with waist/abdominal perimeter and with the homeostatic model assessment of insulin resistance (HOMA-IR) [46]. Moreover, gut permeability was also positively associated with visceral adiposity in healthy overweight women, but in advanced age, as measured by the sucralose/mannitol excretion ratio [47] (Table 1). Altogether these studies point to a relationship between whole gut permeability and visceral adiposity. A study by Brignardello et al. challenged this hypothesis, as no alterations in small intestine barrier permeability (through lactulose/mannitol excretion) in obese versus lean individuals were reported [48]. This discrepancy may arise from the inclusion of males in this study, as age and body mass index (BMI) were comparable to the study conducted by Teixeira et al*.* [46]. As aforementioned in pre-clinical studies, HFD seems to have a preponderant role in barrier impairment (Table 1) and, in the covered human studies, no data regarding dietary habits was evaluated, nor provided. Nonetheless, in healthy subjects, a five-day HFD regimen induced a marked increase in *Firmicutes* and *Proteobacteria* [49]. On the other hand, higher fiber intake was related to lower amounts of *Proteobacteria*, increased SCFA, and weight loss in overweight and obese subjects [50]. A very low-calorie diet (800 kcal/day) induced a marked amelioration of glycemic profile while decreasing *Proteobacteria* and improving gut barrier integrity, in obese women, as measured by lactulose/mannitol and polyethylene glycol excretion and by plasma zonulin levels [51] (Table 1). To the best of our knowledge, no studies in patients with obesity were conducted with more precise techniques that would allow visual detection of GI abnormalities, such as confocal endomicroscopy, a technique allowing in vivo imaging of GI lesions and structures, using a contrast agent during endoscopy, nor using in vitro assessment of intestinal biopsies. In animal models, GI integrity assessment by confocal endomicroscopy has been used in rats and mice to evaluate the gastric and colonic walls [52, 53], but no studies in obesity models were reported to date.

Altogether, these data show the powerful role of the diet itself in the fast modulation of gut microbiota and barrier permeability. Some dietary factors have been linked to a balanced microbiome and restored gut function, such as fiber and polyunsaturated fatty acids. Dietary interventions can be designed with greater precision now that the contribution of specific foods on gut microbiota and modulation of enterotypes and inflammation is starting to be unraveled [54]. Both bamboo fiber and omega-3 supplementation in HFD-fed mice restored the *Firmicutes/Bacteroidetes* ratio, and increased SCFAs production (by increasing *Prevotella* and *Bacteroides*), thus being associated with better metabolic outcomes [55, 56]. Cereals, vegetables, fish, and nuts are highly associated with microbiota balance, reduced inflammation, and increased SCFAs production in humans [57], highlighting the potential of healthy dieting in the management of intestinal health. On the other hand, environmental factors such as air pollutants, heavy metals and pesticides, can also shape the gut microbiota, by entering the food chain, thus contaminating aliments [58]. Indeed, microplastics, derived from the degradation of plastic litter, which accumulate in the soil, water and air, have been shown to promote gut dysbiosis in C57BL/6 J mice and alter the fecal BA profile and liver function [59]. Further, traffic-related air pollution is emerging as a possible inductor of gut dysbiosis in rodents [60] and humans [61], and correlates positively with the development of obesity from a young age [62], raising serious concerns about their negative impacts on metabolic health.

Arising as beneficial gut microbiota modulators, probiotic supplementation with *Lactobacillus* spp. has been shown as a promising strategy to counteract intestinal inflammation and barrier disruption, while reverting obesity in mice [8, 63]. Exercise training is also a potent modulator of the gut microbiota, by decreasing the *Firmicutes/Bacteroidetes* ratio and improving intestinal inflammation in sedentary adults with obesity [64]. By reshaping gut microbiota and alleviating inflammation and barrier disruption, these strategies will end up improving overall gut function, metabolic endotoxemia and peripheral tissue’s function, such as adipose tissue and the liver, constituting valuable weapons against metabolic diseases and related gut malfunction.

(2) Consequences of dysbiosis and inflammation on gut function

*The role of gut microbiota on regulating gut endocrine and nervous functions*: the gut communicates with other organs either through nervous connections or endocrine signaling, in an intricate manner, to regulate energy balance [2]. Vagus nerve projections onto the nucleus tractus solitarius regulate food intake, inducing a positive response upon activation of epinephrine neurons co-expressing neuropeptide Y (NPY), or instead, inhibiting it, through activation of norepinephrine ones [65]. Moreover, the vagus-mediated gut-brain-liver connection controls glucose homeostasis by suppressing hepatic glucose production upon nutrient sensing in the duodenum-jejunum [66]. Additionally, the GI tract secretes a myriad of hormones, including the glucagon-like peptide 1 (GLP-1), peptide YY (PYY), glucose-dependent insulinotropic polypeptide (GIP), cholecystokinin (CCK) and ghrelin [67]. While nutrient digestion and absorption stimulate the secretion of anorexigenic signal—GLP-1, CCK and PYY–, nutrient shortage triggers ghrelin secretion from the stomach to promote feeding instead [67]. The synchronicity between neuronal and humoral signals traveling across the crossroads of the gut-brain axis will govern overall energy homeostasis.

A healthy microbiota regulates gut signals secretion by the production of SCFAs from dietary fiber digestion [14]. Within the gut, SCFAs, but mainly butyrate, stimulate GLP-1 and PYY secretion from L-cells, through a G protein-coupled receptor 43 (GPR43)-dependent mechanism [68, 69] (Fig. 1(2)), This favors an anorexigenic and incretin-stimulatory environment that might account for the beneficial metabolic effects. Secondary BA were also shown to stimulate GLP-1 secretion in vitro in enteroendocrine cell lines [70]. Other metabolites derived from bacterial metabolism, such as indole, are stimulants of gut hormones secretion. Indole is derived from bacteria-mediated tryptophan metabolism [71]. Acute indole action (5 min) stimulated GLP-1 secretion from GLUTag cells, by inhibiting voltage-gated K^+^ channels [71]. However, longer exposure to indole (30–240 min) induced a reduction of GLP-1 secretion rate, through a reduction of adenosine triphosphate production in GLUTag cells [71], showing that indole production by bacteria can have a major impact on host metabolism. Whether SCFAs, indole, or other bacterial metabolites can modulate the secretion of other gut hormones, such as CCK or ghrelin, remains yet to discover.

Despite not being able to contact directly with vagal afferent fibers, the gut microbiota produce several neurotransmitters—acetylcholine, dopamine, serotonin, γ-aminobutyric acid —, that can act either in the enteric nervous system or in brain centers through the vagal afferents or via circulation [72] (Fig. 1(2)). Additionally, butyrate was shown to induce a direct stimulatory effect on the vagus nerve in rats, independently of enteric neurotransmitters release/activation, but the mechanisms were not further investigated [73] (Fig. 1(2)).

*Gut dysbiosis, inflammation and impaired gut endocrine and nervous functions:* gut hormones secretion is highly dependent on diet composition (reviewed by Martin et al.) [74]. Diet-induced obesity, accelerated by HFDs, is a major deregulator of the gut-brain axis and gut inflammation has deleterious effects on the overall GI function [74]. Obesity is characterized by alterations in the gut hormones secretion profile, as shown by the decrease in GLP-1 and PYY post-prandial excursions, as well as impaired total and acylated ghrelin levels, which levels remain higher in the post-prandial state when compared to healthy lean controls [67]. Furthermore, obesity is linked to the desensitization of satiety signals in vagal afferent neurons, favoring constitutive orexigenic activity [75].

SCFAs production is also hampered in the context of obesity, as dysbiosis induces the loss of butyrate-producing bacteria [76]. Consequently, the SCFAs-mediated gut hormones secretion, such as GLP-1, may be reduced (Fig. 1(2)). *Lactobacillus* reduction in HFD-fed mice was associated with decreased GLP-1 responsiveness [77]. Moreover, circadian alterations of ileal microbiota were shown to regulate GLP-1 production in L cells, through ileum clock genes regulation [78].

Loss in SCFAs production might also impair the vagal tone since butyrate contributes to vagal afferents stimulation [73] (Fig. 1(2)). Indeed, germ-free mice conventionalized with microbiota from HFD-fed mice displayed a reduction in vagal innervation to the brain [79]. In rats, inducing dysbiosis by an HFD/high-sugar regimen, induces vagal innervation withdrawal at the gut and nucleus solitary tract, dampening the gut-brain axis [32]. Intestinal dysbiosis was also associated with alterations in serotonin availability, through serotonin transporter modulation [80]. Excessive reuptake of serotonin will induce precocious termination of its physiologic effects, impairing normal gut-brain connectivity.

The relationship between the compromise on gut endocrine function and vagal activation in scenarios of intestinal inflammation is yet poorly understood. Nonetheless, colonic GLP-1 and circulating GLP-1 and GLP-2 levels were decreased under inflammatory conditions, in rodent models and patients with inflammatory bowel disease (IBD) [81, 82]. Interestingly, promising data from pre-clinical studies support a protective role of GLPs in the amelioration of IBD, as agonism of their receptors was shown to alleviate colitis, by reducing the expression of inflammatory cytokines [83]. Clinical pilot studies using the GLP-2 analog teduglutide have demonstrated higher and quicker remission rates in Chron’s disease patients, than placebo-treated ones [84]. In a recent study, in patients with IBD, GLP-1-based therapies showed a decreased risk of hospitalization or need for a TNFα inhibitor treatment, compared to patients on other medications [85]. Similarly, plasma PYY levels, colonic PYY and oxyntomodulin-positive cells are decreased in IBD patients [86]. In turn, ghrelin levels were reported to be increased in patients with Crohn’s disease and ulcerative colitis [87]. Altogether, although the crosslink between gut inflammation and altered gut hormones secretion patterns is still not clear, the available data supports the notion that gut inflammatory milieu potentiates the impairment of neuroendocrine mechanisms involved in nutrient-sensing and energy balance. Future studies should address the mechanisms involved in gut hormone dysregulation by inflammation.

The vagus nerve is a major regulator of gut inflammation. In animal models of colitis, vagal nerve stimulation prevents disease worsening, through acetylcholine-mediated anti-inflammatory effects on macrophages [88]. Individuals who underwent vagotomy have an increased risk of developing IBD, highlighting the immunomodulatory effect of the vagus nerve activity [89]. Further, intestinal inflammation is characterized by an increase in serotonin and NPY circulating levels, neurotransmitters with relevant action within the enteric nervous system and in the gut-brain axis [90, 91]. NPY is abundantly expressed in the enteric nerves and plays a role in colonic relaxation. *Npy* knock-out mice were shown to be more protected from the development of dextran sodium sulfate-induced colitis [92]. Colitis induces a major upregulation of enteric neuronal NPY, which triggers an oxidative and inflammatory response through neuronal nitric oxide synthase activation [93]. The NPY system seems particularly relevant as a modulator of inflammatory activity, since NPY receptor 1 antagonism reduced inflammatory cytokines in the gut of an IBD rodent model [93]. Colonic serotonin is increased in IBD patients and colitis rodent models, and inhibition of serotonin synthesis is a sufficient factor to reduce experimental colitis severity [94].

Prebiotics are indigestible fermented foods, frequently used to restore balance in the gut flora, by increasing the amount of beneficial *Bifidobacterium* and *Lactobacillus* spp., and thus of SCFAs [95]. Modulation of the gut microbiota has shown beneficial effects on intestinal function, thus restoring normal gut hormones secretion. Prebiotic supplementation, with oligofructose, ameliorated gut inflammation and intestinal barrier integrity, while restoring GLP-1 and GLP-2 levels, through increased L cells density [95, 96]. Further, pre and probiotics supplementation has marked effects in AT metabolism [95], highlighting a relevant link between the gut and the AT, possibly mediated by both gut hormones and microbiota.

(3) The involvement of gut dysbiosis and endotoxemia in AT dysfunction

*Dysbiosis/inflammation-mediated changes of gut-AT crosstalk:* Under physiological conditions, the gut hormones act on and modulate AT function, to regulate lipid metabolism (GLP-1 stimulates lipolysis, CCK and ghrelin stimulate lipogenesis), insulin sensitivity and glucose uptake (GLP-1), and tissue plasticity (GLP-1 and ghrelin) [67]. This intricate communication is a central piece in the whole-body energy balance regulation, allowing the AT reservoirs to behave accordingly to the energetic status, i.e., post-absorptive or post-prandial. Given that the secretion pattern of gut hormones is altered in obesity, this may be an important contributing factor to AT dysfunction, a major hallmark of both obesity and the metabolic syndrome [67]. Also, as covered above, gut dysbiosis and possible inflammation have deleterious effects not only on endocrine, but also nervous-mediated gut communication, which inevitably ends up by compromising other organs’ function. However, the real contribution of gut dysbiosis/inflammation for compromising gut endocrine and nervous functions is not yet established, neither is the role of the dietary signals for such relationship. One may note that many of the mechanisms and signals governing gut hormone secretion are not known, and only recently their direct action in AT has been disclosed in more detail (reviewed by Rosendo-Silva) [67]. It may be expectable that impaired GLP-1 secretion following loss of SCFA-producing bacteria could have a negative impact on AT plasticity and glucose metabolism, given the known GLP-1 actions in adipose fat [67, 97]. However, the impact of other gut signals is yet to be disclosed.

*The direct contribution of gut signals to AT dysfunction in obesity:* A reduction in the SCFA-producing bacteria, in the context of HFD-driven dysbiosis, is also associated with an increased prevalence of bacteria able to convert other food molecules (such as aromatic amino acids and histidine, among others) in metabolites (namely indoles, cresols, and imidazole propionate) that can affect metabolic function. For instance, this has been observed regarding glucose tolerance and insulin resistance, contributing also to an increase in overall pro-inflammatory effects [98–100]. In fact, while the symbiotic SCFA-producing bacteria contribute to metabolic homeostasis and gut barrier health, the prevalence of pathogenic bacteria contribute to metabolic deregulation and gut barrier impairment, on one side by losing the panoply of protective effects of SCFAs (including the secretion of mucin by goblet cells), and on the other side by presenting a pro-inflammatory profile in opposition to the anti-inflammatory effects of symbiotic bacteria [15, 16]. Gut-derived metabolites can directly impair the behavior of several extra-intestinal sites, such as the AT, especially the mesenteric one due to the close location, the liver, the pancreas, and the skeletal muscle [101]. Upon intestinal barrier leakiness, the whole organism is exposed to the metabolic by-products of the local microbiota (Fig. 1(3)). Some of these metabolites are important metabolic mediators, including SCFAs, H_2_S, p-cresol, and trimethylamine [100]. Namely, they display relevant effects in adipocytes. While SCFAs and H_2_S induce antilipolytic effects and favor lipid storage [102, 103], 4-cresol and secondary bile acids induce lipolysis [104]. Nonetheless, other microbiota-derived factors, can trigger harmful events in extra-intestinal organs. A well-known example is LPS, responsible for metabolic endotoxemia, giving rise to chronic low-grade inflammation, characteristic of obesity and metabolic syndrome [21]. By activating the TLR4/MD-2 complex in target organs, and after cluster of differentiation 14 (CD14)-dependent internalization, LPS-mediated signaling triggers the production of several pro-inflammatory cytokines, similar to its role in mediating gut inflammation [105]. The primary site of action of LPS is suspected to be the liver, through the portal vein circulation [106]. In fact, hepatocytes and Kupffer cells highly express CD14 [107]. LPS migration through portal circulation is mainly mediated by high-density lipoprotein (HDL), specifically HDL_3_, which paradoxically plays a protective role against LPS-mediated liver injury [106]. Still to uncover is how HDL_3_ suppresses LPS bioactivity, possibly by inducing its clearance, and if and how this mechanism is impaired in obesity, which is usually associated with alterations in HDL cholesterol levels and properties. Indeed, a recent study reported increased HDL_3_ levels in patients with obesity, but LPS levels were not simultaneously assayed [108]. Circulating LPS has been suggested as a biomarker of MAFLD [109]. Indeed, TLR4 activation is required for fructose-induced experimental MAFLD in mice, highlighting the role of diet-driven dysbiosis as a fuel for metabolic diseases [110]. Similarly, the secondary BA deoxycholic acid can cause liver injury, by activation of the inflammasome [111].

Circulating LPS eventually reaches other peripheral organs besides the liver, such as AT. The relevance of LPS signaling on AT emerged from the early studies revealing increasing adiposity as one of the main outcomes of metabolic endotoxemia [23]. In fact, human AT macrophages highly express the TLR4/CD14 machinery, and so do adipocytes, although to a lower extent [112]. LPS can modulate several AT events—inflammation, matrix remodeling/plasticity, metabolic activity—possibly being involved in tissue dysfunction (Fig. 1(3)). Subcutaneous AT from patients with obesity displayed a marked increase in the LPS-signaling machinery and LPS-treated adipocytes initiated an immunogenic response, relying on NF-ĸB and the inflammasome [113]. Accordingly, NLRP3 silencing prevented LPS-induced inflammation in human visceral adipocytes [114]. Vatier et al*.* reported higher sensitivity of subcutaneous, rather than visceral adipocytes, to the deleterious effects of LPS, with lower amount of LPS triggering a more powerful immune response in abdominal subcutaneous AT explants [115]. Metabolic endotoxemia impairs the AT matrix remodeling by inducing fibrosis both in vivo and ex vivo, through a transforming growth factor beta 1 (TGFβ1)-dependent mechanism [101]. LPS is also able to directly stimulate the lipolytic activity of AT, inducing hormone-sensitive lipase phosphorylation (S^650^), thus increasing serum fatty acids levels [115, 116]. It has also been shown that adiponectin gene expression was downregulated in 3T3-L1 cells after LPS treatment, and so was the adiponectin receptor in muscle cells [117]. Moreover, adiponectin suppressed LPS-induced NF-ĸB activation, and consequent inflammation, in pig subcutaneous adipocytes and 3T3-L1 cells [118]. Given that circulating adiponectin is decreased in subjects with obesity, as young as 5 years of age, and in individuals with the metabolic syndrome [118, 119], this may increase the susceptibility of AT to LPS-mediated deleterious effects. Low-dose of LPS infusion (600 µg  kg/day) during 4 weeks in C57BL/6N mice induced insulin resistance, as shown by an aberrant increased insulin secretion without proportional clearance of plasma glucose levels [120]. Co-administration of the anti-inflammatory resveratrol, restored normal insulinogenic index, suggesting inflammation of insulin-dependent organs to be the cause of LPS-mediated impaired glucose homeostasis [120]. However, Stevens et al*.* demonstrated that an acute low-dose LPS (0.1 µg/kg) had a positive impact in glucose tolerance in C57BL/6N mice, but without increasing glucose uptake [121]. Thus, for further accuracy, it is important to reflect on the definition of high and low LPS dosages. LPS levels in circulation fluctuate along the day (from 1 to 50 pg/mL) [122], increasing after a single high-fat meal, even in lean healthy subjects [123].

Although controversial at the beginning, the number of reports on bacterial DNA and bacteria itself in the blood has been drastically increasing, leaving no doubt as to the existence of the blood microbiome. In healthy individuals, the blood-borne bacteria resemble that of the skin and mouth, substantially different from intestinal microbiota [124]. Remarkably, in patients with obesity and T2DM, the blood microbiota might be more closely related to that of the intestines [125, 126]. In fact, *Escherichia–Shigella* is increased in plasma and stool samples from patients with T2DM [127], highlighting once again the involvement of gut bacteria and respective metabolites on the pathophysiology of obesity and related metabolic disorders. The idea of blood-resident bacteria, with no association with an ill state or infection, needs further reflection, as it could lead to the discernment between healthy versus unhealthy blood microbiome, and thus, be a useful biomarker aiding the prediction or diagnosis of a specific disease.

More recently, and with the advances in sequencing techniques, the easiness to dig deeper into human physiology from a microbial perspective allowed ground-breaking discoveries. Today, we acknowledge our “microbial-selves”, as referred by Castillo et al*.,* not only because our intestines house more bacteria than our body houses cells, but also, since apparently several organs have a resident microbiota [124, 128]. Major players in the pathophysiology of obesity and metabolic syndrome—the liver, AT depots (mesenteric, omental and subcutaneous), skeletal muscle and the pancreas -, have been described to harbor their own microbiota (reviewed by Massier et al.) [128, 129]. The “tissue microbiota hypothesis”, as first raised by Burcelin et al*.* postulates that gut-derived bacteria colonize extra-intestinal tissues, modulating their function and causing the onset of metabolic diseases [130]. The first evidence arrived from pre-clinical studies of Rémy Burcelin and co-workers. HFD-induced obese mice showed translocation of gut-derived live bacteria to blood and mesenteric AT [131]. However, studies with human AT samples, were very controverse at the beginning. In 2016, the first study testing the hypothesis of the tissue microbiota in humans, failed to identify endogenous bacterial DNA in subcutaneous and visceral AT [132]. Nonetheless, in the following year, two studies reported successful characterization of bacterial DNA in human AT [133, 134]. *Ralstonia* spp. (Gram-) was enriched in the mesenteric AT from subjects with obesity. Despite lacking appropriate control conditions, the occurrence of contamination may be discarded, as subcutaneous and omental AT did not yield 16S rRNA amplicons determined by pyrosequencing [134]. Pedicino et al*.* also showed the presence of bacterial DNA in epicardial AT from patients with acute coronary syndrome, but not in control subjects, which was positively associated with inflammasome activation [133]. More recently, in 2020, studies employing the Illumina sequencing technology have confirmed the hypothesis of AT being colonized with microbiota [127, 135]. Being aware of possible sample contamination as a confounding factor, Anhê et al*.* designed an elegant study, considering a set of controls, from the operating room to sample manipulation in the laboratory [127]. Successful 16S rRNA quantification and sequencing, in different AT depots, revealed higher bacterial DNA deposition in the omental AT. However, the mesenteric AT bacterial signature presented the closest resemblance to the gut microbiota, suggesting the gut to be the main source of bacterial DNA to the surrounding AT [127]. Interestingly, the mesenteric AT microbiota, was substantially different between individuals with obesity, with or without T2DM [127], suggesting a close relationship between extra-intestinal bacterial colonization and dysglicemia. More compelling evidence arose from the work of Massier et al. who employed a protocol, enabling to detect live bacteria in human subcutaneous AT of patients with obesity [135]. Findings regarding bacteria compartmentalization and quantity in various AT depots were according to those aforementioned [127]. The bacterial load in omental AT was highly correlated with macrophage infiltration and inflammatory genes, in obese subjects with T2DM. Despite being merely associative, these data advert a possible involvement of the tissue microbiota in the development of local inflammation. Further, HOMA-IR provided good explanatory power on Bray–Curtis dissimilarity analysis, in all fat depots (subcutaneous, omental, and mesenteric), suggesting an interplay between dysglycemia and shifted bacteria signatures in obesity [135].

Nonetheless, some important questions remain to be answered: (1) how to disclose the gut as the main source of extra-intestinal bacterial colonization? As discussed above, in vivo evaluation of intestinal barrier integrity is challenging, especially in humans. Nonetheless, the link between metabolic endotoxemia, obesity and intestinal permeability must be further investigated by employing more reliable techniques and finding new biomarkers of the leaky gut to correlate with in vivo tests, especially since increased intestinal permeability is also a hallmark of other obesity-related metabolic diseases, such as MAFLD and T2DM. (2) When does AT depots colonization takes place? The difficulty to include normal-weighted subjects in these studies has led to uncertainty regarding this matter, since the doubt persists on whether AT colonization exclusively happens during chronic inflammatory diseases, such as obesity and related T2DM. Further, the aforementioned studies only focused on the obesity scenario with or without T2DM. However, one must interpret the metabolic sequelae of obesity as a spectrum, ranging from metabolically healthy obesity (normoglycemia and insulin sensitivity) to the metabolically unhealthy phenotype, comprising the consequent ill-stages of insulin resistance, progressive dysglycemia and established T2DM. Hence, future studies should address the chronological timepoint of AT bacterial colonization and how it responds to the consequent metabolic dysregulation that may follow obesity; (3) what are the implications of AT colonization in tissue function? Doubt remains on what could be the influence of a given bacteria signature on AT function. Existing data focused only on AT inflammation and relied on associative links, derived from correlation analyses, that do not necessarily reflect causality. More studies are needed to unravel the contribution of specific bacteria to the modulation of AT (dys)function. In particular, the reshaping of resident and infiltrating AT immune cells population may arise as a determinant factor for the development of a pro-inflammatory environment that may trigger insulin resistance and dysmetabolism.

## Conclusion

Many of the components of the diet (fibers, macro, and micronutrients) are digested by the microbiota and most of the physiological functions of the gut are regulated by the metabolites resulting from such modifications. Westernized diets with a lipid and sugar overload, and potentially also contaminated with residual levels of chemicals and pollutants, induce rapid and marked dysbiosis (alterations in gut microbiota composition and its metabolites), that may be accompanied by gut inflammation and barrier disruption. While the role of Westernized diets in inducing dysbiosis is overall consistent (increasing the *Firmicutes/Bacteroidetes* ratio) (Table 1), in rodents and humans, the development of gut inflammation is a more complex process to decipher, as there is conflicting evidence regarding a pro-inflammatory response (Table 1). Despite some studies showing increased colonic inflammatory markers upon HF or high-sugar diet consumption in rodents, there is a lack of observation of histological features of inflammation, even when diets were taken to an extreme duration (Table 1) [28]. However, this does not exclude that a chronic low-grade inflammation might be taking place in the colon, resulting from increased levels of pro-inflammatory factors. Nevertheless, studies with germ-free mice have shown the central role of microbiota in Westernized diet-induced gut inflammation [34]. Slight alterations in the microbiota (at family or genus level), possibly attributed to differences in diet composition, might be the explanation for the discrepancy in the inflammatory response.

The gut microbiota exerts a modulatory effect on vagal tone and the production of GLP-1, PYY, serotonin, mainly through SCFAs, having a major impact in gut-brain communication (Fig. 1(2)). Diet-induced dysbiosis was shown to hamper gut hormones production and to cause vagal withdrawal in rodents, although their consequences in the secretion of gut neuroendocrine factors are still unknown [31, 73, 75]. However, it is necessary to depict the involved triggering agents, from specific bacteria to inflammatory markers, as this investigation is still in its first steps. Thus, there is still a long way to understand the relation between gut microbiota and impaired energy balance, at central and peripheral levels.

Westernized diets consumption and metabolic diseases, such as obesity, MAFLD and T2DM, are associated with the development of chronic low-grade inflammation [17, 136]. Intriguingly, this feature is also present in patients with T2DM without obesity and some patients with obesity do not have increased markers of systemic inflammation, raising questions about the mechanisms linking metabolically (un)healthy obesity with gut dysbiosis/endotoxemia. The diet-induced modulation on gut microbiota is associated with increased intestinal permeability and endotoxemia (Table 1), which can be the initial trigger for low-grade inflammation by activating TLR4 in the liver and adipose tissue (Fig. 1(3)). Moreover, the extravasation of bacteria itself to the blood and tissues is arising as a putative modulator of inflammatory pathways. Despite their role in the establishment of low-grade inflammation is also still to unravel, this highlights the importance of diet manipulation as an important factor not only to ameliorate gut dysbiosis, but also in regulating pathogenic bacteria and metabolites translocation to the adipose tissue.

## Data Availability

Data sharing is not applicable to this article as no data sets were generated or analysed during the current study.
